# Transactions of the Medical and Physical Society of Calcutta. Vol. VIII. Part I

**Published:** 1838-10-01

**Authors:** 


### Transactions of the Medical and Physical Society of Calcutta. Vol. VIII. Part I.

[Concluded.]

We have already laid before our readers a more or less complete account of several of the papers in this volume. We shall now present a sketch of the contents of such of the remainder as are adapted for our purpose. We feel great pleasure in bearing testimony to the zeal and ability of our fellow labourers in the East. The march of medical science is as evident in India as in Europe.

### I. Case of Ranula in which the Left Submaxillary Gland was extirpated; with Remarks. By *J. G. Malcolmson,* Esq. Assistant Surgeon, Madras Establishment.

In the beginning of 1828, a sickly-looking Hindoo boy, nine years of age, was brought to Chicacole from an unhealthy hill district, on account of a swelling which extended from one ear to the other, over the angles of the jaw and to the sternum, near which it was more distended than above, and slightly pendulous, so as to admit of being raised from the skin covering the superior extremity of the bone; it was quite soft, and evidently contained a fluid. The disease commenced about a year before, below the jaw and a little to the left of the chin, and had gradually increased downwards, and up to the ears. The patient stated, that for some time before the appearance of the tumor, there had been a discharge of pus half an inch behind, and lateral of the mouth of the duct of the left submaxillary gland, where there was a depressed cicatrix, about a line in diameter, on the formation of which the swelling commenced. Near the cicatrix, there was a carious tooth. A very slight discharge of saliva from the left submaxillary duct could still be observed. There was no swelling in the mouth; but a hardness could be felt below the jaw, a little to the left side, and there were several scars in the centre of the swelling, caused by repeated applications of the actual cautery. Mr. Malcolmson concluded that the submaxillary ducts or gland having been inflamed, probably from the irritation of the diseased tooth, the passage of the saliva was interrupted, and making its way into the loose cellular substance of the throat, had gradually distended the integuments.

A puncture was made into the most prominent part of the swelling, and twenty ounces of a glairy transparent fluid, of a light brown tinge were discharged, as well as a hard substance resulting from the inspissation of the liquid. The opening was then left free, and the discharge carefully pressed out every morning. Twice, however, the wound closed, and a new puncture was necessary. On the last occasion, a tent was left in the wound, some inflammation followed, and a good deal of matter was discharged along with the glairy fluid; the skin contracted a little, but it was evident, that little could be be expected from this plan.

" Having procured a very fine probe, I found it passed into the duct of the gland, and some saliva flowed from it: this diminished my confidence in the opinion I had formed of the disease, but convinced me, that it could not be cured by endeavouring to restore the communication by the mouth, and removing the swelling by puncture and pressure. I therefore resolved to remove a portion of skin constituting the front of the sac, ascertain if the fluid came from the gland, and take such farther measures as might be necessary.

On the 30th, I placed him on a table, and punctured the tumor three inches above the sternum; and after the fluid was evacuated, cut away an oval portion of the skin (of the size of 2½ by 2 inches), which was much thickened from the cicatrices of the cauteries and punctures. The throat now exhibited an extraordinary appearance: from behind the ears, over the angles of the jaw, and down to the chest, it seemed as if carefully dissected; the blue veins and parotid glands shining through the cellular membrane. I in vain looked for any opening, from which the fluid might come; the possibility of its being derived from the left parotid, or of its being an encysted tumor, whose sac had become condensed with the surrounding parts, at the same time occurred to my mind; in either of which cases nothing more remained, than to close up the wound, and to try what could be done by pressure. The cellular membrane in the mesial line had become condensed, and formed the hardness felt in the neck;—it was therefore removed.

Having examined with attention a soft round body, of the size and colour of a small lymphatic gland, partly embedded in the left submaxillary gland, I observed a very minute puncture, as if from the point of a needle, and on gentle pressure, a glairy fluid flowed from it. I immediately proceeded to separate the gland from the surrounding parts, which at first was not difficult; but on getting into the hollow of the jaw, it was more firmly attached, and the space was so narrow, that it was difficult to use the knife. A ligature was passed through the gland, by which it was drawn out; but it was still difficult to tie the vessels which bled. One very considerable vessel, (the lower maxillary,) was cut, the bleeding from which was stopt by pressure made on the carotid; but it could not be secured, until Lieut. H., (who in the absence of another surgeon assisted), put his thumb into the mouth, and pushed the gland downwards, which greatly assisted the rest of the operation. Then by passing a curved needle through the parts several times, and cutting between the gland and the ligature, the whole was removed, except a small process, which passed between the anterior belly of the digastric and the mylo-hyoideus muscles, and probably joined the sublingual gland; to this the actual cautery was applied." 18.

The edges of the wound were brought together by ligatures and plaister, and the greater part of it healed readily. But an abscess formed at the lower part of the left side of the neck—the cicatrix in the mouth opened and discharged matter—the abscess in the neck itself was opened—the latter wound closed, and the opening in the mouth closed afterwards—and in less than a month from the time of performing the operation he was quite well. The cicatrix in his throat was very small, and did not disfigure him; the opening into the mouth had healed, and a minute portion of saliva flowed from the duct of the submaxillary gland, on the left side, probably from the sub-lingual gland.

Mr. Malcolmson has related this case, principally for the purpose of shewing that, in extreme cases, extirpation of the sub-maxillary gland may not only be practicable but beneficial. It is pretty clear that the whole gland was not extirpated, yet the result appears to have been as favourable as could be desired.

It has been frequently observed, that, although ranula is believed to be dilatation of the duct of the sub-maxillary gland, anatomical proof of the fact is defective. Mr. Malcolmson observes:—

"In my patient, the origin of the tumor was clearly seen; the small blue gland-like body, from a very minute opening in which, the saliva passed into the cellular substance of the throat, was a small cyst, into which some branches of the duct of the gland were seen to run. I endeavoured to leave it unbroken, so as to have an opportunity of demonstrating this fact; but its coats being very thin, it was broken during the removal of the gland. Dupuytren refers to Jean Murimcks, as having thought he had demonstrated, that it depended on an accumulation of saliva in the ducts, which open under the tongue. That this is the origin of the tumors which appear in the mouth is pretty certain, and the peculiarity of the present case is explained, by the fact of one or more of the small ducts having been obliterated in a situation distant from the mouth. So small a tube, as that which was closed up in this patient, could not admit of distention to the enormous size the swelling attained; accordingly, a very minute opening, (not larger than would be caused by the finest needle-point,) was formed, and the gradual distention of the integuments followed in consequence." 22.

### II. A Case of Ulcerated Stricture of the Œsophagus, communicating with the Trachea. By A. K. Lindesay, Esq.

Sergeant J. C., æt. 55, was admitted into hospital in June, having fallen some weeks before, when intoxicated, and felt as if his chest had struck on a sharp hard body. From the time of the fall, he has had slight wandering pain in upper part of the right side of the thorax, and has gradually lost the power of swallowing solid food. No disease of the heart, lungs, or ribs, could be detected. The pain was speedily removed by leeches, blisters, and attention to the general health; but the difficult deglutition remained unmitigated; the smallest tube of a stomach-pump could be passed into the stomach, giving a feeling of continued resistance for an inch or so, about opposite to the top of the sternum; the orifice of the tube on withdrawal was always filled with thick puriform mucus, streaked with blood. He was benefited by passing the tube at intervals for some little time after he left the hospital, but about the end of August he returned, stating that he felt increasing difficulty in taking food: he had lived for some time on bread reduced to a pulp in milk, which he was obliged to swallow very slowly. Two attempts on different days were unsuccessful with the tube formerly introduced; on the third day, Aug. 30, an elastic catheter was made to pass the stricture after using considerable force; the end of the catheter, when the point was fairly engaged in the thickened part, was just within the teeth; satisfied with this for the day, the stiletto of a small cathether was fitted with a bit of sponge, and slightly curved, laterally, so that on introducing it, the sponge protruded at the eye of the instrument, and next day, Sept. 1, a weak solution of nitrate of silver was applied by the sponge, and a little thrown into the catheter, while withdrawing it through the structure; no bad effect followed this attempt, at medication.

This plan was persevered with for upwards of a fortnight, but, at the end of that time, no instrument could be passed, it appearing to hitch on a fold. On the 2d of October, a catheter could again be got to pass, and (to attempt dilatation of the stricture from below, as recommended by Fletcher of Gloster), a piece of gut was fitted on a catheter, introduced flaccid, and by blowing made as large as a walnut: after inflation, it was easily withdrawn. It was now noticed, that there were difficulties to the passing of an instrument, before reaching the former seat of stricture.

After this, he gradually got worse, and became troubled with a cough, hawking up glairy pus and blood.

Nov. 26.—"Yesterday, about 11 a.m., during a violent fit of coughing, he threw up a mass of coagulated blood, (as he described it, though it was probably a slough), and since that time, has not been able to swallow a drop of liquid. I introduced a catheter, which passed *into* the thickened parts, *not beyond*; injected a little water to relieve thirst, if possible, but it was *blown* back, with much coughing and spitting of bloody mucus: so that now there is evidently a communication between the trachea and œsophagus." 43.

Every attempt to swallow caused violent coughing. Nutritive enemata, &c. were ordered.

On the 27th, an attempt to pass the tube was unsuccessful. On the 28th, the tube, after much difficulty passed through, twice. After this, the tube was passed frequently, sometimes with more, sometimes with less difficulty. Vomiting became more and more frequent and distressing, the symptoms of gastritis supervened, and on the 14th of December the patient died.

*Dissection.*—Extreme emaciation. Marks of recent inflammation of pleuræ and lungs.

Mucous membrane of the stomach inflamed—the same with the colon as low as the middle of the sigmoid flexure.

The pharynx was vascular, and covered with green mucus. There were found no marks of disease in the larynx or front of the trachea: at its sides, and extending around the œsophagus to the adjacent portion of the arch, and descending aorta, there was considerable increase of substance; the aorta was quite healthy, adhering by its outer coat to the thickened œsophagus; on cutting out a portion of the trachea in front, an opening was observed communicating with the œsophagus, irregularly circular, about the area of a shilling, and situated about half way between the cricoid cartilage and the bifurcation; the mucous membrane of the trachea, for some distance above and below the aperture, was vascular, and covered with purulent mucus; tracing the œsophagus from the cardiac extremity, by slitting it, the lining membrane was found vascular, but not decidedly diseased until just above the bifurcation of the trachea, where it was found thickened and ulcerated in its whole circumference: the ulcer occupied fully four inches of the canal; it was covered by thick pus, and was of a very irregular surface, there being several pale lumpy projections, and a few specks, which felt rough and cartilaginous; the canal above the aperture was much more capacious than below, but even downward, it easily admitted the little finger to pass.

The case is interesting, because it shews the inutility, we would say, the injuriousness, of persevering with instruments in cases of malignant or ulcerated disease of the œsophagus. We do not refer to this case in particular, but to the general fact. The present case appears to have been treated with great skill and judgment.

III. Case of extensive Liver Abscess, successfully opened. By Archibald Colquhoun, Assistant Surgeon, 12th N. I.

B. M'Grath had been in hospital under different medical officers from the 30th of January till the 23d of May, 1835, when Mr. Colquhoun took charge of the artillery. He was then in a state of great debility, with pulse very small and quick, pain in the right side, increased on pressure, or on taking a full inspiration. Tongue and skin natural, bowels generally confined. Leeches were frequently applied to the side, after which a caustic blister. Purgatives were daily given, and ij. gr. of calomel every night in hopes of inducing ptyalism. But no sensible effect of this kind was induced. About the 6th of May, fulness and prominence of the side first became distinct, after which it increased daily, under the application of large poultices and dry cupping-glasses. On the 15th, Mr. Colquhoun performed the operation in the presence of Mr. Savers, now member of the Medical Board, and Mr. Jackson, Surgeon of the 8th Cavalry.

"An incision about two inches long, immediately under the edge of the ribs, was made into the prominent part of the swelling, down to the peritoneum, on cutting through which with an abscess lancet, a copious discharge of purulent matter of a reddish yellow colour, took place: in the course of half an hour seven pints were discharged. The incision was dressed with simple ointment; he expressed immediate relief from the pain, and could then lie down comfortably, having been obliged to sleep in a sitting posture for some time before. At 9, p. m. I was suddenly called to see him, and found him cold, pulseless, and nearly dead. Owing to hæmorrhage from some of the vessels of the abdominal parietes, upwards of two pounds of coagulated blood was lying under him. Pressure with a pledget of ol. terebinth. on lint immediately stopped the bleeding." 157.

The bleeding did not recur—pus was discharged from the wound in some quantities, up to the 23d inst., from which time he continued to improve slowly till about the beginning of June, when a troublesome diarrhœa came on. But this was checked, he returned to his duty in August, and, in the cold season, Mr. C. saw him marching in perfect health and as stout as ever, through Allahabad.

IV. Dislocation of the Os Humeri reduced after a Month and Four Days.

The subject of the accident was a native, 30 years of age. The head of the os humeri was distinctly felt in the axilla; the limb had acquired some degree of mobility, and the arm could be nearly approximated to the side. Several attempts having failed, the pulleys at last effected the reduction.

V. Operation performed in Persia, for the Removal of Opacity of the Cornea. By S. M. Griffiths, Esq.

"In this part of Persia (Tehran) an operation is practised for the cure of opacity of the cornea, which may be worthy of the attention of the members of the medical and physical society of Calcutta, as it is said to be frequently successful in improving the transparency of the cornea, if not always capable of restoring perfect vision. The object of this operation seems to be, to completely cut off the vascular communication, by excision of a circular portion of the conjunctiva at a small distance from the margin of the cornea, which is accomplished by fixing eight small hooks into the conjunctiva, about a line from the union of the cornea with the sclerotica, quite round the cornea; the operator then raises that part of the conjunctiva by pulling these hooks towards him, and with a pair of scissors, he cuts off the portion thus raised, and completely insulates the conjunctiva covering the cornea, the consequence of which is the gradual absorption of the opacity of the part affected, and the cornea recovers its transparency. The after-treatment is very simple, consisting merely in the introduction of a small quantity of antimony between the lids; in fact, the result of the operation is confidently expected to be successful without any other application."—*Appendix*, xx.

